# Contraceptive, condom and dual method use at last coitus among perinatally and horizontally HIV-infected young women in Atlanta, Georgia

**DOI:** 10.1371/journal.pone.0202946

**Published:** 2018-09-12

**Authors:** Lisa B. Haddad, Jennifer L. Brown, Caroline King, Nicole K. Gause, Sarah Cordes, Rana Chakraborty, Athena P. Kourtis

**Affiliations:** 1 Department of Gynecology & Obstetrics, Emory University School of Medicine, Atlanta, GA, United States of America; 2 Department of Psychiatry & Behavioral Neuroscience, University of Cincinnati College of Medicine, Cincinnati, OH, United States of America; 3 Division of Reproductive Health, National Center for Chronic Disease Prevention and Health Promotion, Centers for Disease Control and Prevention, Atlanta, GA, United States of America; 4 Department of Psychology, University of Cincinnati, Cincinnati, OH, United States of America; 5 Department of Pediatrics, Division of Infectious Diseases, Emory University School of Medicine, Atlanta, GA, United States of America; Massachusetts General Hospital, UNITED STATES

## Abstract

**Objective:**

To evaluate factors within the social-ecological framework associated with most or moderately effective contraception, condom and dual method use at last coitus among young, HIV-infected women in Atlanta.

**Methods:**

This is a cross-sectional study conducted from November, 2013 until August, 2015 at the Grady Infectious Disease Clinic in Atlanta, Georgia. We recruited perinatally and horizontally HIV-infected women of ages 14–30 years to complete an audio computer-assisted self-interview. We evaluated factors within a social-ecological framework associated with most or moderately effective contraceptive use (hormonal contraception or an IUD), condom use, and dual method use (use of condom and most or moderately effective contraceptive) at last coitus.

**Results:**

Of 103 women enrolled, 74 reported a history of sexual activity. The average age was 22.1; 89% were African American, 52% were perinatally infected, 89% received combination antiretroviral therapy, and 63% had undetectable viral loads. At last coitus, 46% reported most or moderately effective contraception, 62% reported condom use and 27% reporting dual-method use. The odds of most or moderately effective contraceptive use was significantly reduced among those with detectable viral loads (versus undetectable viral loads; aOR 0.13 [0.04, 0.38]). Older age (aOR 0.85 [0.74, 0.98] and more frequent coitus (>once/week versus < = once/week; aOR 0.24 [0.08, 0.72]) was significantly associated with reduced condom use. Having a detectable viral load (versus undetectable viral loads; aOR 0.13 [0.03, 0.69]) and more frequent coitus (>once/week versus < = once/week; aOR 0.14 [0.03,0.82]), was associated with reduced dual method use, while being enrolled in school (aOR 5.63 [1.53, 20.71]) was significantly associated with increased dual method use.

**Conclusions:**

Most or moderately effective contraception, condom and dual method use remained inadequate in this cohort of young HIV-infected women. Individual-level interventions are needed to increase the uptake of dual methods with user-independent contraceptives.

## Introduction

HIV-infected adolescent and young adult women experience high rates of unintended pregnancies, sexually transmitted infections (STI), and secondary HIV transmission to uninfected partners[1–3]. Furthermore, unintended pregnancies increase perinatal HIV transmission risk to offspring.[2] The risk of HIV infection among young women is heightened in the Southeastern United States, and specifically Georgia (GA), where the burden of HIV/AIDS falls disproportionately upon African American youth[4, 5]. While teen pregnancy rates are dropping in Georgia, rates remain consistently above the national average[6, 7]. The United States (U.S.) National Strategy for HIV/AIDS[8] and the Healthy People 2020 Objectives[9] aim to reduce both unintended pregnancies and STI/HIV among vulnerable populations; to achieve this goal, it is critical to understand and encourage improvement of reproductive health behaviors, practices and associated outcomes in young HIV-infected women.

Currently, there are limited data on contraceptive practices, sexual behaviors, knowledge, and attitudes regarding pregnancy and STI/HIV prevention among young HIV-infected women. Most research has focused either on younger adults not in the United States[10–15] or on older HIV-infected adults in the United States[16, 17]. However, the challenges, beliefs and practices of younger HIV-infected women likely differ from those in other countries and those of older adult counterparts. Studies of uninfected adolescents and young adults may not be truly representative since reproductive health and STI prevention priorities may change with HIV infection, reinforcing the importance of condom use.[18] Reports on younger HIV-infected individuals often lack documentation of patient beliefs on contraceptive methods and how this may influence choice and use.[17, 19, 20]

HIV-infected women engaging in unprotected sex risk secondary HIV transmission to uninfected partners and acquisition of other STIs or super-infection with drug-resistant HIV.[1, 3, 21, 22] Previous data demonstrate high rates (40–63%) of unprotected sex among young HIV-infected women. [18] Furthermore, qualitative data highlight differing attitudes among horizontally and perinatally infected young women; while some perinatally infected young women insist on consistent condom use, many are challenged with HIV serostatus disclosure and communication about barrier protection, so they defer condom use.[23] Clinical guidelines recommend comprehensive reproductive health counseling to include dual methods of pregnancy prevention by highly effective contraceptive methods, including contraceptive implants and intrauterine devices (IUD), and STI/HIV prevention through consistent use of condoms.[24, 25] While current Centers for Disease Control and Prevention (CDC) medical eligibility guidelines do not restrict contraceptive use based on HIV status alone,[26] many providers may be unaware of these recommendations. Further, may providers worry about drug-drug interactions between hormonal contraceptives and antiretroviral therapy (ART), lack training in contraceptive provision, or believe that effective hormonal contraception will lead to reduced condom use. Thus, it is unclear what information young HIV-infected women are receiving and how these messages inform contraceptive choices and sexual behaviors; it is also unclear how these issues may differ between women who were perinatally versus horizontally HIV infected.

This study aimed to explore current reproductive health knowledge, attitudes and practices among HIV-infected adolescents and young adults receiving medical care at an HIV clinic in Atlanta, GA. Additionally, we explored factors associated with contraception, condom and dual-method use at last coitus within a social ecological framework to determine the possible correlates of less effective contraceptive practices to address in future reproductive health interventions. Expanding our understanding of contributors to sexual behaviors and family planning practices within a social ecological framework can inform future efforts to improve preventive care in this high-risk population.

## Materials and methods

### Study population and procedures

This is a cross-sectional study of HIV-infected, female patients attending a comprehensive pediatric and adolescent HIV clinic and a women’s HIV clinic in Atlanta, Georgia. Participants receiving care at this clinic have different types of insurance coverage with cost varying based on insurance coverage. Contraceptive pills and the injectable depot medroxyprogesterone acetate (DMPA) were available on site every day. A gynecologist was available one clinic day per month for contraceptive services, consultation and to provide long-acting reversible contraceptive methods on site. Additionally, women could be referred to a title X clinic which was about 1 mile away for free contraceptive provision or may choose to see their gynecologist independently to receive contraceptive services. We obtained a partial HIPAA waiver to review the daily clinic schedule to identify potential participants who were women within our inclusion criteria age range. All potential participants were approached by a research assistant (RA) in the clinic waiting room or were provided with a flyer with study information. For individuals interested in participating, the RA or research staff member escorted the patient to a private room, read a recruitment script, answered questions about the study, and assessed study eligibility. Women were eligible if they were 1) receiving care at either of the HIV clinics, 2) female, 3) aged 14–30 years, and 4) able to read English. Individuals were excluded if they were currently pregnant or incarcerated. Eligible individuals provided written informed consent. The study was conducted from November, 2013 until August, 2015; 155 patients were approached: 19 patients did not meet eligibility criteria (12%), 29 women declined participation (19%) and 107 completed an audio computer-assisted self-interview (ACASI) (69%). Of the 107, 4 women had inconsistent survey findings (reported prior pregnancy and reported no history of sex); thus 103 had complete data available for this analysis. The study procedures were approved by the Emory University Institutional Review Board (IRB), the CDC IRB, and the Grady Research Oversight Committee.

Participants completed a 30-minute ACASI survey assessing their contraceptive practices, sexual behaviors, and knowledge, attitudes, and beliefs regarding pregnancy and STI/HIV prevention. Additionally, their medical charts were reviewed to abstract information on most recent HIV viral load and CD4+ T-cell count as well as STIs diagnosed within the last year. Participants received a $25 gift card for completing the ACASI.

### Study outcomes

We chose not to limit inclusion to the study based on prior sexual activity as among this age group, initiation of sex is not always a specifically planned event and adolescent health providers are often aiming to prepare young women for their initial sexual encounter. However, for our condom use and contraceptive analysis, only women who were sexually active (i.e., responded “yes” to the question, “Have you ever had vaginal sex?”) were included in the analyses (n = 74); we excluded women who were not sexually active (i.e., responded “no” to the question, “Have you ever had vaginal sex?”; n = 29) from analyses. For the descriptive analyses, those who reported a history of any sexual activity (n = 74) were categorized according to contraceptive use at last coitus to create four outcome variables as follows: (a) condom use only (yes/no); (b) most or moderately effective contraception use (hormonal contraception method or IUD) only (yes/no); (c) dual method use (condom and most or moderately effective contraceptive use; yes/no); or (d) no method use (yes/no condom or most or moderately effective contraceptive use at last coitus). These groups were then separately evaluated as 3 different method groups: (1) those who used most or moderately effective contraception (b + c / a + d); (2) those who used condoms (a + c / b+ d); (3) those who used dual methods (c / a + b + d). Thus, women who reported dual method use contributed to the numerator (“yes”) of the three method outcomes (condom use, most or moderately effective contraception use, and dual method use), whereas for all four variables all sexually active participants were included in the denominator.

### Potential correlate measures evaluated

**Fig 1:**
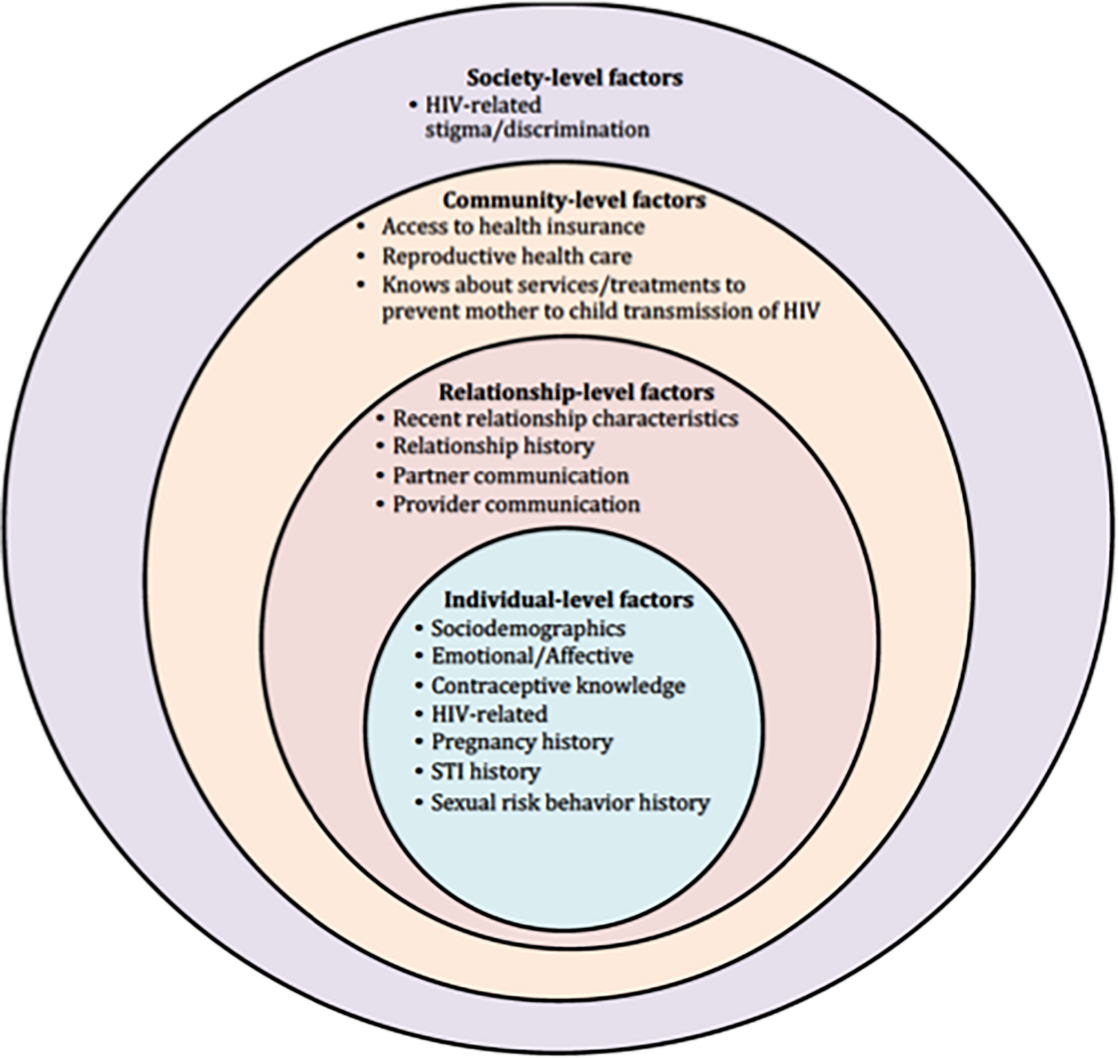
Social-ecological model utilized in this study for evaluating most or moderately effective contraception use, condoms use and dual method use.

Potential correlates of the method use outcomes (condom use, most or moderately effective contraception use, and dual method use) were categorized into 4 domains of the social-ecological model (see Fig 1): Individual-Level. Relationship-Level Factors, Community-Level and Society-Level Factors. See appendix for details regarding specific factors evaluated.

### Data analytic approach

Data were analyzed using SAS Version 9.4 (SAS Institute, Cary NC). Simple logistic regression models were used to determine univariate associations between potential correlates and each of the three contraception-related outcome variables of interest. For continuous variables, non-linear associations were explored through the evaluation of each variable as categorical with breaks at median and quartiles as well as categorical groups commonly used in the literature. If no clear non-linear association was present, variables were maintained as continuous variables in the model. Factors associated with an outcome at the p < .10 level were included in the stepwise multivariate logistic regression model for the corresponding outcome variable. Models were inspected for multi-collinearity based on a VIF greater than or equal to 10; variables with a VIF > 10 were excluded from the model. As such, the variable “has children” (Yes/No) was excluded from the models predicting condom use at last coitus and dual protection at last coitus. Adjusted odds ratios and corresponding 95% confidence intervals were generated for the factors retained in the stepwise regression model for each outcome variable.

## Results

Of the 103 women who completed the survey, 52.4% were perinatally infected and 28.2% reported no prior sexual intercourse (Table 1). Among the 54 perinatally infected women, 22 reported no prior sexual activity (40.1%). Average age was 22 (range 14–30) years; 89.3% were African American, 41.8% were enrolled in school, 39.8% had at least one prior pregnancy, and 89.3% were taking medication for HIV (combination ART). The majority (63.1%) had an undetectable viral load, and the mean CD4+ T-cell count was 446.0 cells/μl (+/- 289.53), with 43.7% having a CD4+ T-cell count of >500 cells/μl.

**Table 1:** Description of individual-level factors among young HIV-infected study participants and association between these factors and most or moderately effective contraception use only, condom use only, and dual method use at last coitus.

Variable	Total n = 103	Not sexually active n = 29	Most or moderately effective contraceptive use only n = 14	Condom use only n = 26	Dual method use n = 20	No method at last sex n = 14	Most or moderately effective contraceptive use versus not using most or moderately effective contraceptive	Condom use versus no condom	Dual method use versus non dual method use
							Unadjusted OR (95%CI)	Unadjusted OR (95%CI)	Unadjusted OR (95%CI)
***SOCIODEMOGRAPHIC CHARACTERISTICS***									
Age *mean (SD)*	22.1 (4.4)	18.4 (3.3)	23.4 (3.6)	23.7 (4.1)	21.3 (3.3)	26.6 (3.0)	0.83 (0.73–0.95)***	0.85 (0.75–0.97)**	0.79 (0.68–0.93)***
Race									
*Black*	92 (89.3)	27 (93.1)	13 (92.9)	26 (100)	16 (80.0)	10 (71.4)	1(Ref)	1(Ref)	1(Ref)
*White*	5 (4.9)	0 (0)	0 (0)	0 (0)	2 (10.0)	3 (21.4)	0.83 (0.13–5.29)	0.37 (0.06–2.35)	2.04 (0.31–13.33)
*Other*	6 (5.8)	2 (6.9)	1 (7.1)	0 (0)	2 (10.0)	1 (7.1)	3.72 (0.37–37.72)	0.55 (0.07–4.15)	3.06 (0.40–23.54)
Hispanic	5 (4.9)	1 (3.5)	1 (7.1)	1 (3.9)	2 (10.0)	0 (0)	3.77 (0.37–38.09)	1.89 (0.19–19.03)	2.89 (0.38–22.04)
Enrolled in school	43 (41.8)	21 (72.4)	3 (21.4)	5 (19.2)	12 (60.0)	2 (14.3)	3.72 (1.29–10.74)**	2.70 (0.87–8.41)*	6.60 (2.14–20.39)****
Ever homeless	18 (17.5)	2 (6.9)	4 (28.6)	5 (19.2)	3 (15.0)	4 (28.6)	0.89 (0.29–2.72)	0.53 (0.17–1.61)	0.56 (0.14,2.21)
Currently employed	30 (29.1)	5 (17.2)	8 (57.1)	5 (19.2)	9 (45.0)	3 (21.4)	4.00 (1.43–11.15)***	0.68 (0.25–1.81)	1.94 (0.68–5.59)
***EMOTIONAL/ AFFECTIVE***									
Existential Wellbeing *mean (SD)*	45.9 (9.1)	46.4 (8.9)	49.3 (8.8)	42.8 (8.4)	46.9 (8.7)	46.1 (10.9)	1.10 (1.00–1.11)*	0.96 (0.91–1.01)	1.02 (0.96–1.08)
CES-D Score *mean (SD*)	5.8 (6.0)	4.8 (5.3)	3.6 (3.3)	7.4 (6.8)	5.7 (7.2)	7.3 (6.1)	0.93 (0.86–1.01)*	1.03 (0.96–1.12)	0.98 (0.90–1.07)
***CONTRACEPTIVE KNOWLEDGE***									
Knowledge Score *mean (SD)*	6.5 (1.6)	6.0 (1.7)	7.2 (1.9)	6.3 (1.3)	7.1 (1.2)	6.5 (1.6)	1.46 (1.03–2.08)**	0.89 (0.65–1.23)	1.26 (0.87–1.83)
***HIV-RELATED***									
Taking HIV meds	92 (89.3)	29 (100.0)	10 (71.4)	23 (88.5)	17 (85.0)	13 (92.9)	0.43 (0.11–1.61)	1.45 (0.40–5.28)	1.00 (0.23–4.15)
Current viral load									
*Undetectable*	65 (63.1)	22 (75.9)	10 (71.4)	9 (34.6)	18 (90.0)	6 (42.9)	1 (ref)	1 (ref)	1 (ref)
*Detectable*	38 (36.9)	7 (24.1)	4 (28.6)	17 (65.4)	2 (10.0)	8 (57.1)	0.13 (0.04–0.38)****	0.94 (0.36–2.43)	0.10 (0.20–0.45)***
CD4 Count *mean (SD)*	446.0 (289.5)	519.6 (249.4)	553.9 (154.2)	306.4 (299.6)	516.7 (345.6)	343.6 (264.83)	1.00 (1.00–1.01)***	0.99 (0.99–1.00)	1.001 (1.00–1.003)*
Hospitalized for HIV related illness	29 (28.2)	6 (20.7)	2 (14.3)	9 (34.6)	6 (30.0)	6 (42.9)	0.51 (0.19–1.42)	1.21 (0.43–3.37)	0.93 (0.31–2.85)
Percentage of meds taken in last month									
*≥90%*	48 (46.6)	9 (31.0)	7 (50.0)	15 (57.7)	7 (35.0)	10 (71.4)	1 (ref)	1 (ref)	1 (ref)
*<90%*	55 (53.4)	20 (69.0)	7 (50.0)	11 (42.3)	13 (65.0)	4 (28.6)	2.38 (0.3–6.07)*	1.69 (0.65–4.38)	2.70 (0.93–7.85)*
Infection route									
*Horizontal*	49 (47.6)	7 (24.1)	8 (57.1)	13 (50.0)	10 (50.0)	11 (78.6)	0.75 (0.30–1.89)	0.47 (0.18–1.26)	0.69 (0.25–1.93)
*Perinatal*	54 (52.4)	22 (75.9)	6 (42.9)	13 (50.0)	10 (50.0)	3 (21.4)	1 (ref)	1 (ref)	1 (ref)
***PREGNANCY HISTORY***									
Prior pregnancy	41 (39.8)	1 (3.5)	10 (71.4)	15 (57.7)	6 (30.0)	9 (64.3)	0.59 (0.24–1.49)	0.40 (0.15–1.06)*	0.25 (0.08–0.76)**
Has children	36 (48.7)	**-**	9 (64.3)	12 (46.2)	6 (30.0)	9 (64.3)	0.71 (0.29–1.79)	0.36 (0.14–0.95)**	0.34 (0.12–1.03)*
Primary caregiver for children	32 (43.2)	**-**	9 (64.3)	10 (38.5)	5 (25.0)	8 (57.1)	0.86 (0.34–2.16)	0.31 (0.12–0.83)**	0.33 (0.11–1.05)*
Want a baby together in the next year§ (Yes vs no/not sure)	14 (19.4)	3(21.4)	2(18.2)	1(4.6)	3(18.8)	5(55.6)	0.95 (0.25–3.54)	0.22 (0.06–0.87)**	0.98 (0.23–4.28)
***STD HISTORY***									
STD diagnosed within the year	23 (22.3)	3 (10.3)	4 (28.6)	7 (26.9)	7 (35.0)	2 (14.3)	1.65 (0.59–4.63)	1.60 (0.53–4.82)	1.70 (0.56–5.16)
***SEXUAL RISK BEHAVIOR HISTORY***									
Ever had anal sex	27 (36.5)	-	8 (57.1)	6 (23.1)	6 (30.0)	7 (50.0)	1.45 (0.56–3.76)	0.31 (0.11, 0.83)**	0.67 (0.22–2.03)

§ among those in a with a boyfriend in the last 12 months; STD = Sexually transmitted disease; SD = Standard Deviation; OR = Odds Ratio; CI = Confidence Interval. P-value for Chi-square * p < .10- ** p < .05- *** p < .01- **** p < .001

### Contraceptive use and knowledge

Among those with prior sexual activity (n = 74), 14 (18.9%) reported most or moderately effective contraceptive use only, 26 (35.1%) reported condom use only, 20 (27.0%) reported dual method use, and 14 (18.9%) used no method at last coitus. While slightly over half (51.5%) had heard of the levonorgestrel IUD (Lng-IUD; Mirena or Liletta), fewer had heard of the copper IUD (Cu-IUD; Paragard, 30.1%) or etonogestrel implant (Eng-Implant; Implanon or Nexplanon, 32.0%). Most women had received some most or moderately effective contraception in the past, primarily DMPA (63.1%) and oral contraceptive pills (42.7%); 9.7%, 8.7% and 2.9% had a lifetime history of ever using the Lng-IUD, Eng-Implant and Cu-IUD, respectively. The mean contraceptive knowledge score for the sample was 6.5 (possible range 0–9).

### Factors associated with most or moderately effective contraceptive use at last coitus

Almost half (45.9%) of the women with a history of sexual activity used some contraceptive method, either most or moderately effective form of birth control at last coitus (Tables 1–4). In univariate analyses, being younger (p < 0.01), enrolled in school (p < 0.05), employed (p < 0.01), or having a higher total contraceptive knowledge score (p < 0.05), an undetectable HIV viral load (p < 0.001), and a higher CD4+ T-cell count (p < 0.01) were associated with increased odds of most or moderately effective contraceptive use at last coitus. In the multivariable analysis (Table 5), only HIV viral load remained associated with most or moderately effective contraceptive use where those with detectable viral loads (versus undetectable viral loads) had lower odds of most or moderately effective contraceptive use (aOR 0.13, 95% CI [0.04, 0.38]).

**Table 2:** Description of relationship-level factors among young HIV-infected study participants and association between these factors and most or moderately effective contraception use only, condom use only, and dual method use at last coitus.

Variable	Total n = 103	Not sexually active n = 29	Most or moderately effective contraceptive use only n = 14	Condom use only n = 26	Dual method use n = 20	No method at last sex n = 14	Most or moderately effective contraceptive use versus not using most or moderately effective contraceptive	Condom use versus no condom	Dual method use versus non dual method use
							Unadjusted OR (95%CI)	Unadjusted OR (95%CI)	Unadjusted OR (95%CI)
***CURRENT/ MOST RECENT RELATIONSHIP***									
Had a boyfriend in the previous 12 months	**72 (70%)**	**14 (48.3%)**	**11 (84.6)**	**22 (84.6)**	**16 (80.0)**	**9 (75.0)**	1.02 (0.30–3.40)	1.19 (0.34–4.11)	0.86 (0.23–3.18)
Is/was partner a lot older that you?	31 (43.1)	4 (28.6)	6 (54.6)	12 (54.6)	6/(37.5)	3 (33.3)	0.85 (0.30–2.40)	1.10 (0.37–3.26)	0.6 (0.19–1.95)
Description of relationship now§									
*Boyfriend/ex-bf*	51 (70.83)	8/(57.1)	10 (90.9)	16 (72.7)	12 (75.0)	5 (55.6)	1 (ref)	1 (ref)	1 (ref)
*Casual partner (on/off bf; no one special; friend)*	21 (29.2)	6 (42.9)	1 (9.1)	6 (27.3)	4 (25.0)	4(44.4)	0.48 (0.14–1.63)	1.07 (0.31–3.72)	0.94 (0.25–3.53)
Was he STD or HIV tested while having sex together § (Yes vs no/do not know)	50 (67.6)	-	7(50.0)	16 (61.5)	16 (80.0)	11 (78.6)	1.01 (0.38–2.67)	1.27 (0.47–3.44)	2.35 (0.69–8.03)
Do you think your most recent partner has sex with others§ (Yes/not sure vs no)	17 (41.5)	-	4(40.0)€	5(50.0)€	5 (41.7)€	3 (33.33) €	0.95 (0.27–3.31)	1.43 (0.41–5.01)	1.01 (0.26–3.96)
How often do you and he have sex									
*Once per week or less*	51 (68.9) *	-	5 (35.7)	19 (73.1)	18 (90.0)	9 (64.3)	1 (ref)	1 (ref)	1 (ref)
*More than once per week*	23 (31.1)	-	9 (64.3)	7 (26.9)	2 (10.0)	5 (35.7)	1.12 (0.42–2.99)	0.24 (0.09–0.69)***	0.18 (0.04–0.83)**
***RELATIONSHIP HISTORY***									
More than 1 partner in past 6 months	7 (9.72)	-	0 (0.0) €	2 (7.69)	3 (15.00)	2 (15.38)€	0.91 (0.19–4.38)	1.40 (0.25–7.81)	2.07 (0.42–10.24)
More than 3 lifetime partners	43 (58.1)	-	5 (35.7)	16 (61.5)	13 (65.0)	9 (64.3)	0.68 (0.27–1.71)	1.71 (0.66–4.42)	1.49 (0.51–4.31)
Number of partners since HIV-infected *mean (SD)*	3.24 (4.7)	2.88 (4.8)	2.86 (2.8)	3.04 (3.1)	4.35 (7.8)	2.64 (2.6)	1.04 (0.94–1.16)	1.05 (0.92–1.19)	1.06 (0.95–1.18)
***PARTNER COMMUNICATION***									
Talked about pregnancy	41 (55.4)	-	8 (57.1)	9 (34.6)	15 (75.0)	9 (64.3)	2.56 (0.99–6.61)*	0.71 (0.27–1.83)	3.23 (1.03–10.15)**
Talked about condom use	35 (47.3)	-	7 (50.0)	11 (42.3)	12 (60.0)	5 (35.7)	1.90 (0.75–4.80)	1.33 (0.52–3.43)	2.02 (0.71–5.75)
Talked about contraceptive use	23 (31.1)	-	5 (35.7)	7 (26.9)	8 (40.0)	3 (21.4)	1.90 (0.69–5.02)	1.21 (0.43–3.37)	1.73 (0.59–5.08)
Talked about STD testing	27 (36.5)	-	4 (28.6)	9 (34.6)	7 (35.0)	7 (50.0)	0.72 (0.28–1.87)	0.82 (0.31–2.18)	0.92 (0.31–2.67)
Talked about HIV testing	38 (51.4)	-	10 (71.4)	12 (46.2)	9 (45.0)	7 (50.0)	1.4 (0.56–3.51)	0.54 (0.21–1.41)	0.71 (0.25–1.98)
***PROVIDER COMMUNICATION***									
"Have you ever had a discussion about reproductive health or family planning with a health care worker?"	46 (44.7)	13 (44.8)	8 (57.1)	9 (34.6)	9 (45.0)	7 (50.0)	1.5 (0.60–3.78)	0.56 (0.22–1.44)	1.02 (0.37–2.87)

€ 1 or more missing; § among those in a with a boyfriend in the last 12 months;; STD = Sexually transmitted disease; SD = Standard Deviation; OR = Odds Ratio; CI = Confidence Interval. P-value for Chi-square * p < .10- ** p < .05- *** p < .01- **** p < .001

**Table 3:** Description of community-level factors among young HIV-infected study participants and association between these factors and most or moderately effective contraception use only, condom use only, and dual method use at last coitus.

Variable	Total n = 103	Not sexually active n = 29	Most or moderately effective contraceptive use only n = 14	Condom use only n = 26	Dual method use n = 20	No method at last sex n = 14	Most or moderately effective contraceptive use versus not using most or moderately effective contraceptive	Condom use versus no condom	Dual method use versus non dual method use
							Unadjusted OR (95%CI)	Unadjusted OR (95%CI)	Unadjusted OR (95%CI)
***COMMUNITY***									
Access to health insurance									
*No*	36 (35.0)	6 (20.7)	7 (50.0)	10 (38.5)	7 (35.0)	6 (42.9)	1 (ref)	1 (ref)	1 (ref)
*Yes- Private*	9 (8.7)	5 (17.2)	0	1 (3.85)	1 (5.0)	2 (14.3)	0.38 (0.04–4.09)	0.77 (0.10–6.18)	1.10 (0.10–12.27)
*Public/other*	58 (56.3)	18 (62.1)	7 (50.0)	15 (57.7)	12 (60.0)	6 (42.9)	1.03 (0.40–2.67)	1.59 (0.60–4.23)	1.41 (0.48–4.16)
"Do you receive reproductive health or OB/GYN services from any other clinic or doctor besides the Ponce Clinic?"	21 (20.4)	3 (10.3)	7 (50.0)	6 (23.1)	2 (10.0)	3 (21.4)	1.24 (0.43–3.59)	0.38 (0.13–1.12)*	0.26 (0.06–1.27)
Knows about services/treatments to prevent mother to child transmission	73 (71.6) *	18 (62.1)	12 (85.7)	21 (80.8)	11 (57.9)€	11 (78.6)	0.58 (0.20–1.68)	0.54 (0.17–1.71)	0.31 (0.10–0.98)*

OB/GYN = Obstetrician Gynecologist; SD = Standard Deviation; OR = Odds Ratio; CI = Confidence Interval.

P-value for Chi-square * p < .10- ** p < .05- *** p < .01- **** p < .001

**Table 4:** Description of society-level factors among young HIV-infected study participants and association between these factors and most or moderately effective contraception use only, condom use only, and dual method use at last coitus.

	Total n = 103	Not sexually active n = 29	Most or moderately effective contraceptive use only n = 14	Condom use only n = 26	Dual method use n = 20	No method at last sex n = 14	Most or moderately effective contraceptive use versus not using most or moderately effective contraceptive	Condom use versus no condom	Dual method use versus non dual method use
***SOCIETY***									
HIV-related stigma/ discrimination (total score)							1.00 (0.95–1.04)	0.99 (0.94–1.04)	1.00 (0.95–1.05)
*mean (SD)*	18.90 (10.1)	18.86 (11.1)	18.36 (15.9)	18.15 (6.4)	18.85 (6.7)	21 (11/63)			

SD = Standard Deviation; OR = Odds Ratio; CI = Confidence Interval.

**Table 5:** Multivariable Models for most or moderately effective contraception use, condom use and dual method use.

Predictor Variable	AOR (95% CI)
***Outcome*: *Most or moderately effective contraceptive use at last coitus***	
***HIV-RELATED***	
Current viral load	
Undetectable	1
Detectable	0.13 (0.04, 0.38)
***Outcome*: *Condom use at last coitus***	
***SOCIODEMOGRAPHICS***	
Age	0.85 (0.74, 0.98)
***CURRENT/ MOST RECENT RELATIONSHIP***	
How often do you and he have sex	
Once per week or less	1
More than once per week	0.24 (0.08, 0.72)
***Outcome*: *Dual method use at last coitus***	
***SOCIODEMOGRAPHICS***	
Enrolled in school	5.63 (1.53,20.71)
***HIV-RELATED***	
Current viral load	
Undetectable	1
Detectable	0.13 (0.03,0.69)
***CURRENT/ MOST RECENT RELATIONSHIP***	
How often do you and he have sex	
Once per week or less	1
More than once per week	0.14 (0.03,0.82)

AOR = adjusted odds ratio from multivariable logistic regression models using stepwise regression. For each of the 3 models, variables with AOR data are the only remaining variables in final models after stepwise elimination. OR are adjusted for the effect of the other variables included in the final model after stepwise elimination.; Y/N = Yes/No; for these variables reference is No. CI = Confidence Interval.

### Factors associated with condom use at last coitus

The majority (62.2%) of women with a history of sexual activity reported using a condom at last coitus (Table 1A–1C). In univariate analyses, younger age (p < 0.05), not having children (p < 0.05), not desiring a baby in the next year (p < 0.05), not being a primary caregiver to children (p < 0.05), not having had anal sex (p < 0.05), and having less frequent coitus (once per week or less vs. more than once per week; p < 0.01) were associated with increased odds of condom use at last coitus. Taking antiretroviral medication, medication adherence, viral load and CD4+ T-cell count were not associated with condom use at last coitus. In the multivariable model (Table 5), older age (aOR 0.85, 95% CI [0.74,0.98]) and more frequent coitus (> once/week versus < = once/week; aOR 0.24, 95% CI [0.08, 0.72]) remained significantly associated with reduced odds of condom use.

### Factors associated with dual method use at last coitus

About a quarter (27.0%) of the women with a history of sexual activity reported using dual methods at last coitus (Table 1A–1C). In univariate analyses, dual method use was significantly associated with increased odds of being younger (p < 0.01), enrolled in school (p < 0.001), having talked to their partner about pregnancy (p < 0.05), having an undetectable HIV viral load (p < 0.01), not having a prior pregnancy (p < 0.05), and less frequent coitus (once per week or less vs. more than once per week; p < 0.05). In the multivariable model (Table 2), being enrolled in school (aOR 5.63; 95% CI [1.53, 20.71]) was associated with increased odds of dual method use, while having a detectable HIV viral load (versus undetectable; aOR 0.13, 95% CI [0.03, 0.69]) and more frequent coitus (> once/week versus < = once/week; aOR 0.14, 95% CI [0.03, 0.82]) remained significantly associated with non-use of dual methods.

## Discussion

Among our cohort of young HIV-infected women in Atlanta, Georgia, ineffective pregnancy prevention and unsafe sexual practices were prevalent, despite participants being actively engaged in comprehensive HIV care. Similar rates of low contraceptive and dual method use have been described in other HIV-infected cohorts[27] and uninfected young African American cohorts.[28] Condoms were the most prevalent form of contraceptive used among women in our cohort, a finding that is reflected in prior studies among HIV-infected women, both domestically and globally.[27, 29] This likely reflects increased concerns regarding HIV-transmission to uninfected partners; however, this approach is suboptimal for prevention of unintended pregnancy. Furthermore, while condom use as a preventive strategy for HIV/STIs is essential, almost 40% of these young, HIV-infected women reported not using a condom at last coitus.

There have been several studies of behavioral interventions involving counseling or education aimed at increasing condom or dual method use uptake and continuation among young women living with HIV [30, 31], however few have demonstrated efficacy. Many of the factors associated with use of condoms, most or moderately effective contraception, and dual methods were at an individual or relationship level, compared to community or societal level. By recognizing that individual-level factors seem to be the strongest influencers of safer sexual practices, efforts towards individually tailored patient-centered prevention counseling may be a particularly important reproductive health intervention.

While partner communication and disclosure are often encouraged as part of counseling for HIV-infected individuals, partner communication factors related to HIV/STI prevention or exclusivity did not appear to influence practices in the cohort. Among sexually active women, those perinatally infected did not behave differently from than their horizontally infected counterparts with regard to pregnancy and HIV/STI prevention. However, those with improved virologic suppression and higher CD4+ T-cell counts were more likely to use contraception. These findings suggest that HIV-infected women who are adherent to antiretrovirals may be more consistent with clinic visits and other medications, including the injectable or oral contraceptive methods. Given that poor virologic control is associated with increased risk of mother-to-child transmission, the need for more effective strategies to address pregnancy prevention is paramount to management in this challenging group. One potential approach is to reduce the user-dependent contraceptives by promoting long-acting reversible contraceptives.

While contraceptive use was higher among women with virologic suppression, condom use did not significantly differ between groups. Although this suggests that knowledge of virologic suppression might not alter condom use, this is a dynamic relationship that will require a longitudinal study design. Similarly concerning is that those with more frequent coitus reported less condom use. This finding has been reported by other investigators with some proposing “condom use fatigue.”[32, 33] Alternatively, factors such as more frequent coitus and older age may reflect a more stable relationship, where condom use may be perceived as less important. While barrier protection is pivotal to STI/HIV prevention, their use remains inadequate, necessitating the investigation of other approaches, including couples counseling and testing, antiretroviral treatment as prevention, pre-exposure prophylaxis of uninfected partners, and utilization of microbicides as they become available within the context of a multi-faceted HIV prevention approach.

One might expect that desire for a child in the next year would be associated with contraceptive use at last coitus. The data did not support this. However, since few women desired children in the next year, the confidence intervals were wide. Contraceptive use is not solely influenced by desire for a child, highlighting the need to recognize the broad cadre of factors that determine usage.[34] Given the high rates of unintended pregnancy, coupled with the risk of mother-to-child transmission, efficacious reproductive health counseling messaging needs to be further developed for HIV-infected young women who do not desire children. It is also important to engage women in fertility discussions within the context of routine HIV care.[35] Safe contraceptive practices should be tailored to an individual’s priorities, which may not be static, especially for younger women. Accordingly, ongoing reproductive health counseling within the context of HIV care is desirable. While we are unaware of proven interventions among HIV-infected young adults in the United States that have increased contraceptive use, research among uninfected women have highlighted that structured counseling, removal of cost and immediate access to highly effective contraception can increase the uptake of highly effective contraceptives and reduce unintended pregnancy[36–39].

This is one of the first studies to examine potential factors that influence contraceptive and condom use practices from a social-ecological framework among a high-risk cohort of young HIV-infected women in the United States. However, there are several limitations for this analysis. While a strength of our study was the broad range of potential influences we evaluated, this can result in a greater chance for at least one Type I error. Further, our small sample size limits our power to detect potential associations that may exist as well as to conduct a multinomial logistic regression to evaluate distinct categorical differences among those who use condoms only, contraceptives only or dual method use. Additionally, as our data are cross-sectional, we cannot comment on causality or temporality of these factors or know if changes in any of these characteristics may result in subsequent changes in practices. We largely relied on self-report, which may increase our chances of recall and social desirability bias. To reduce this risk of social desirability bias, we aimed to ensure confidentiality and utilized ACASI. As we did not assess partner characteristics, such as HIV status, we are limited in our analysis of partner dynamics. Although only 19% of subjects approached declined participation, our results may be biased towards individuals more interested in or knowledgeable about contraception or those with different sexual practices than those who declined participation. Lastly, we focused on a population of young adults in metropolitan Atlanta with HIV, thus generalizability of our findings may be limited to other cohorts of young HIV-infected women.

In conclusion, our results highlight the need to enhance individual-level interventions to improve pregnancy and STI/HIV prevention practices among young HIV-infected women in the United States. While provider-level, societal- and community-level factors may be important to other aspects of an individual’s overall wellbeing, they were not strongly influential on behaviors in our study. Thus, efforts must shift focus to developing and evaluating individual-level interventions, such as patient-tailored education and counseling, to increase the uptake of dual methods with user-independent contraceptives. Furthermore, development of new preventive strategies, such as multipurpose prevention technologies that are effective at preventing pregnancy and STIs and/or HIV, may help to overcome the persistent challenges in consistent condom use.

## Supporting information

S1 File Appendix: Variables evaluated in social ecological model. (DOCX)

S2 File De-identified data from manuscript. (CSV)
