# Supplementary material for: Contraceptive, condom and dual method use at last coitus among perinatally and horizontally HIV-infected young women in Atlanta, Georgia
Source: PLoS One. 2018 Sep 12;13(9):e0202946. doi: 10.1371/journal.pone.0202946 (PMC6135381; doi:10.1371/journal.pone.0202946)
Supplement: S1 File — (DOCX) [file pone.0202946.s001.docx]

**Supporting Information**

**S1. Appendix: Variables evaluated in Social Ecological Model:**

*1. Individual-Level Factors*

*Sociodemographic Characteristics.* Participants reported on the following sociodemographic characteristics: age; race (Black, White, Other); Hispanic (Yes/No); current enrollment in school (Yes/No); current employment (Yes/No); and lifetime history of ever being homeless (Yes/No).

*Emotional/Affective*. Depressive Symptoms were measured with the 8-item Center for Epidemiological Studies-Depression Revised scale(27) (CES-D), which assesses the presence of depressive symptoms in the past seven days. Existential Well-Being was measured with a 10-item subscale measuring existential well-being from the Spiritual Well-Being Scale.(28)

*Contraceptive Knowledge.* Participants responded to nine true/false items related to knowledge of specific contraceptive methods. A total score was calculated indicating the number of correct responses.

*HIV-Related Health Status.* Participants’ medical records were abstracted to ascertain the most recent viral load (coded as detectable vs. undetectable) and CD4+ T-cell count (cells/µl). Participants self-reported their current use of ART (Yes/No); percentage of ART medication taken in the last month (<90% versus >=90%); past hospitalizations for HIV-related illness (Yes/No); and, HIV infection route (perinatal vs. horizontal).

*Pregnancy History.* Participants reported whether they had a prior pregnancy (Yes/No), whether they had children (Yes/No), and whether they were the primary caregiver for a child (Yes/No). Participants also indicated their desire for a baby in the next year (No or Not sure/Yes).

*STI History.* Participants’ medical charts were abstracted to determine if they had been diagnosed with an STI in the last year (Yes/No).

*Sexual Risk Behavior History.* Participants reported their frequency of sexual activity (< once a week vs. ≥ once a week) and whether they had ever engaged in anal sex (Yes/No).

2. *Relationship-Level Factors*

*Current or Most Recent Relationship.* Participants indicated whether or not they had a partner in the last 12 months (Yes/No) and the nature of the relationship (boyfriend or ex-boyfriend/casual partner). Participants indicated whether the partner was “a lot older than them” (Yes/No), whether they believed the most recent partner had sex with others (Yes/No or Not sure), and if the most recent partner got tested for an STD or HIV at any time while they were together and having sex (No or do not know/Yes).

*Relationship History.* Participants reported the number of sexual partners they had (a) in their lifetime (<3, ≥3); (b) in the last 6 months (≤1 vs. >1); and (c) since they became HIV infected.

*Partner Communication.* Participants indicated whether or not they had discussed (a) pregnancy; (b) condom use; (c) contraceptive use; (d) STD testing; (e) HIV testing; and (f) exclusivity in their relationship with their current partner (Yes/No).

*Provider Communication.* Participants indicated whether they had ever discussed family planning or reproductive health with a healthcare provider (Yes/No).

*3. Community-Level Factors*

*Access to Health Insurance.* Participants indicated whether or not they had access to health insurance (Private, Public or other or Don’t Know).

*Reproductive Health Care Access/ Knowledge of services.* Participants indicated whether they received reproductive health services from a provider outside of this clinic (Yes/No). Participants reported if they knew about treatment options to prevent mother-to-child transmission of HIV (Yes/No) and if so if the availability of services/treatments to prevent transmission to child affect desire to have children (Y/N)

*4. Society-Level Factors*

*HIV-Related Stigma and Discrimination Experiences.* Participants responded to 11 items indicating the frequency on a 6-point Likert scale (Never to About Daily) by which they had experienced stigma or discrimination as result of their HIV serostatus.(29) A total score was calculated across the items, with higher scores indicating more frequent experiences of HIV-related discrimination.
